# Inferring cell developmental stage-specific lncRNA regulation in the developing human neocortex with CDSlncR

**DOI:** 10.3389/fnmol.2022.1037565

**Published:** 2023-01-13

**Authors:** Meng Huang, Jiangtao Ma, Junpeng Zhang

**Affiliations:** ^1^Department of Automation, Xiamen University, Xiamen, China; ^2^Department of Computer Science, University of Tsukuba, Tsukuba, Japan; ^3^School of Engineering, Dali University, Dali, China

**Keywords:** single-cell transcriptomics, noncoding RNAs, cell developmental stage-specific lncRNA regulation, lncRNA-target prediction, human neocortex

## Abstract

Noncoding RNAs (ncRNAs) occupy ~98% of the transcriptome in human, and are usually not translated into proteins. Among ncRNAs, long non-coding RNAs (lncRNAs, >200 nucleotides) are important regulators to modulate gene expression, and are involved in many biological processes (e.g., cell development). To study lncRNA regulation, many computational approaches or tools have been proposed by using bulk transcriptomics data. Nevertheless, previous bulk data-driven methods are mostly limited to explore the lncRNA regulation regarding all of cells, instead of the lncRNA regulation specific to cell developmental stages. Fortunately, recent advance in single-cell sequencing data has provided a way to investigate cell developmental stage-specific lncRNA regulation. In this work, we present a novel computational method, CDSlncR (Cell Developmental Stage-specific lncRNA regulation), which combines putative lncRNA-target binding information with single-cell transcriptomics data to infer cell developmental stage-specific lncRNA regulation. For each cell developmental stage, CDSlncR constructs a cell developmental stage-specific lncRNA regulatory network in the cell developmental stage. To illustrate the effectiveness of CDSlncR, we apply CDSlncR into single-cell transcriptomics data of the developing human neocortex for exploring lncRNA regulation across different human neocortex developmental stages. Network analysis shows that the lncRNA regulation is unique in each developmental stage of human neocortex. As a case study, we also perform particular analysis on the cell developmental stage-specific lncRNA regulation related to 18 known lncRNA biomarkers in autism spectrum disorder. Finally, the comparison result indicates that CDSlncR is an effective method for predicting cell developmental stage-specific lncRNA targets. CDSlncR is available at https://github.com/linxi159/CDSlncR.

## 1. Introduction

Long non-coding RNAs (lncRNAs) are a major class of non-coding RNAs with transcript size >200 nucleotides, and lack protein coding capacity (Elling et al., 2016). Unlike small non-coding RNAs (sncRNAs) with strong sequence conservation, lncRNAs has a characteristic of low sequence conservation (Johnsson et al., 2014). Previous studies (Ponting et al., 2009; Pan et al., 2019) have demonstrated that lncRNAs play important roles in many neurobiological processes, e.g., neural development (Sauvageau et al., 2013) and brain disorders (Qureshi et al., 2010; Mishra and Kumar, 2021). In a cell, lncRNAs can regulate gene expression, thereby cause messenger RNA (mRNA) degradation or post-transcriptional repression (Luo et al., 2016), which has significant effects on many critical cellular functions including cell proliferation, cell differentiation, and cell death (Yao et al., 2019). To reveal the biological functions and regulatory mechanisms of lncRNAs, it is necessary to study lncRNA regulation.

Previous studies have shown that lncRNA regulation is tissue-specific (Mattioli et al., 2019), cell-type-specific (Baskozos et al., 2019) and cell developmental stage-specific (Hu et al., 2013). For example, the cell developmental stage-specific lncRNA regulation is closely associated with B-cell development (Dahl et al., 2018). Nevertheless, previous computational methods (Liao et al., 2011; Guo et al., 2015; Wu et al., 2016; Du et al., 2017) using bulk transcriptomics data mostly focus on exploring the lncRNA regulation regarding all of cells, rather than the lncRNA regulation specific to cell developmental stages. This may conceal the heterogeneity of lncRNA regulation across different cell developmental stages. Fortunately, single-cell RNA-sequencing (scRNA-seq) technologies revolutionize the throughput and resolution of bulk RNA sequencing in transcriptomics research (Jaitin et al., 2014; Tal Nawy, 2014), and provides a way to investigate lncRNA regulation at the single-cell or cell-development level.

By using bulk and single-cell RNA sequencing data, several computational methods have been presented for identifying gene regulation at the single-sample and single-cell levels. For example, Liu et al. (2015) and Liu X. et al. (2016) developed two sample-specific gene regulatory network identification methods to construct sample-specific gene regulatory networks from bulk RNA sequencing data. Van de Sande et al. (2020) developed a scalable SCENIC (Single-Cell rEgulatory Network Inference and Clustering) workflow to analyze cell-specific gene regulation from single-cell RNA-sequencing data. Moreover, two cell-specific network identification methods, named CSN (Cell-Specific Network; Dai et al., 2019) and c-CSN (conditional Cell-Specific Network; Li et al., 2021), were also proposed to explore cell-specific transcriptional regulation of genes from single-cell transcriptomics data. Recently, Zhang et al. (2021) proposed CSmiR (Cell-Specific miRNA regulation) to explore cell-specific miRNA regulation from single-cell miRNA-mRNA co-sequencing data. However, the above sample-specific and cell-specific network identification methods are limited to explore transcriptional regulation of genes and miRNA regulation. To understand the heterogeneity of lncRNA regulation across different single-cells or cell developmental stages, it is necessary to infer cell-specific or cell developmental stage-specific lncRNA regulation.

In this work, to understand the heterogeneity of lncRNA regulation across different cell developmental stages, we propose a novel method named CDSlncR (Cell Developmental Stage-specific lncRNA regulation) to infer cell developmental stage-specific lncRNA regulation. CDSlncR has adapted CSN method (Dai et al., 2019) from the following three aspects. Firstly, CSN mainly explore all types of gene–gene interactions from single-cell RNA sequencing data. Given the single-cell RNA sequencing data, CDSlncR extracts expression data of lncRNAs and mRNAs from single-cell RNA sequencing data and then focus on inferring lncRNA-mRNA interactions, rather than all interaction types (including mRNA-mRNA, lncRNA-lncRNA, and lncRNA-mRNA interactions). Secondly, different from CSN (an unsupervised method without using prior knowledge), CDSlncR incorporates putative lncRNA-mRNA interactions as prior knowledge to improve the accuracy of identifying lncRNA-mRNA interactions. Thirdly, CSN is used to identify gene regulation at the resolution of single-cell level rather than cell-development level. To infer cell developmental stage-specific lncRNA regulation, CDSlncR combines the identified cell-specific lncRNA regulatory networks in each cell developmental stage.

Different from other methods (i.e., constructing cell developmental stage-specific lncRNA regulatory networks based on RNA-sequencing data of each cell development stage), CDSlncR firstly infers a lncRNA regulatory network for each cell, and the augmented lncRNA regulatory networks across all of cells in each cell developmental stage are regarded as cell developmental stage-specific lncRNA regulatory networks. To demonstrate the effectiveness of CDSlncR, we apply CDSlncR into single-cell transcriptomics data of the developing human neocortex. Network analysis indicates that the number of predicted and validated lncRNA-mRNA interactions is different across five cell developmental stages of human neocortex, indicating that the lncRNA regulation is unique in each development stage of human neocortex. In addition, we conduct a case study to explore lncRNA regulation of cell-type-specific lncRNA biomarkers. Finally, the comparison result shows that CDSlncR is an effective method for predicting cell developmental stage-specific lncRNA targets, and is helpful for exploring lncRNA regulation in different human neocortex development stages.

## 2. Materials and methods

**Figure 1:**
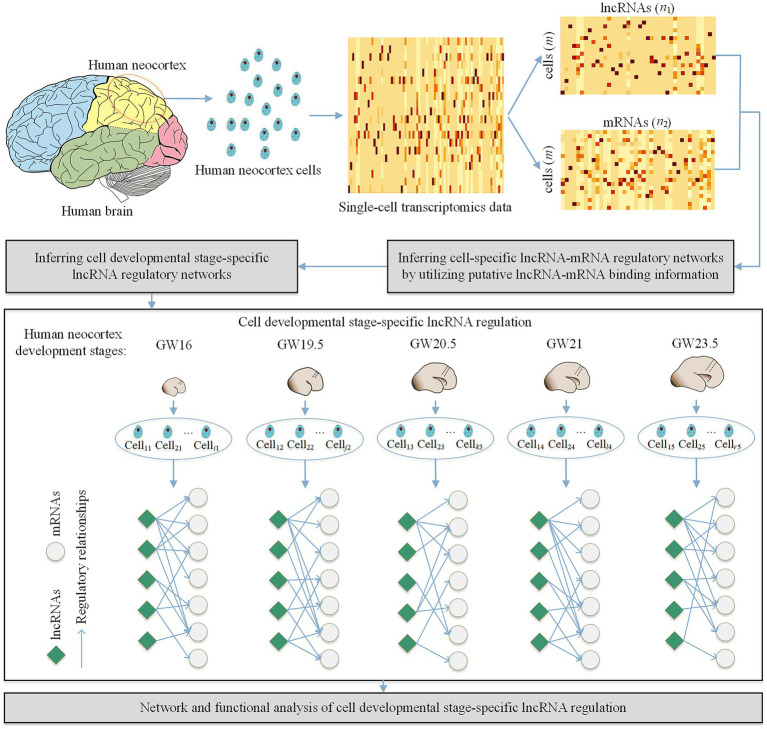
Workflow of CDSlncR. Firstly, we extract the matched lncRNA and mRNA expression data from the single-cell transcriptomics data in the developing human neocortex. Then, we identify m cell-specific lncRNA-mRNA regulatory networks using putative lncRNA-mRNA binding information. Next, we infer cell developmental stage-specific lncRNA regulation based on the identified m cell-specific lncRNA-mRNA regulatory networks. Finally, we conduct network and functional analysis of the identified cell developmental stage-specific lncRNA regulation.

As shown in Figure 1, our proposed method CDSlncR (Cell Developmental Stage-specific lncRNA regulation) includes four components: (i) Single-cell transcriptomics data in the developing human neocortex, (ii) Identifying cell-specific lncRNA-mRNA regulatory networks, (iii) Inferring cell developmental stage-specific lncRNA regulation, and (iv) Network and functional analysis of cell developmental stage-specific lncRNA regulation. In the following, we will describe the four components in detail.

### 2.1. Single-cell transcriptomics data in the developing human neocortex

We obtained single-cell transcriptomics data in the developing human neocortex (including 276 cells and 13,007 genes) from the Gene Expression Omnibus database (the accession number is GSE71315). According to the microdissected radial sections of the tissue at gestational weeks (GW), the dataset contains five development stages of developing human neocortex: GW16, GW19.5, GW20.5, GW21, and GW23.5 (Liu S. et al., 2016). We conducted a gene annotation using HGNC (HUGO Gene Nomenclature Committee^1^) to extract matched lncRNA and mRNA expression from single-cell transcriptomics data. For the duplicate lncRNAs or mRNAs in the dataset, the average expression values of the same gene symbols are computed as the final expression values of them. To consider the nongenetic cell-to-cell variability (Golov et al., 2016), all of lncRNAs and mRNAs with constant expression values are removed. Finally, we used $\log_{2}\left(x+1\right)$ transformation to further normalize the matched lncRNA and mRNA expression data. As a result, we have obtained 247 lncRNAs and 10,208 mRNAs in 276 single-cells from the developing human neocortex. In the developing human neocortex dataset, the number of single-cells in five human neocortex development stages (GW16, GW19.5, GW20.5, GW21, and GW23.5) is 26, 26, 123, 24, and 77, respectively.

### 2.2. Identifying cell-specific lncRNA-mRNA regulatory networks

To construct cell-specific lncRNA-mRNA regulatory networks in human neocortex cells, we use the statistic proposed in (Dai et al., 2019) to measure the association between lncRNAs and mRNAs. For each cell, CDSlncR constructs a lncRNA-mRNA regulatory network. To control the false positives of lncRNA-mRNA interactions, the predicted lncRNA-target interactions from NPInter v4.0 (Teng et al., 2020), ENCORI (Li et al., 2014) and LncRNA2Target (Cheng et al., 2019) are incorporated into CDSlncR.

To evaluate the associations between lncRNAs and mRNAs, we perform the statistical test for each putative lncRNA-mRNA pair ($lncR_{u}$and$mR_{v}$) in a cell. In cell *k*, $u_{k}$ and $v_{k}$ represent expression values of $lncR_{u}$ and $mR_{v}$, respectively. $m_{u}^{\left(k\right)},$ $m_{v}^{\left(k\right)}$ and $m_{\mathrm{uv}}^{\left(k\right)}$ denote the number of cells in the neighborhoods of $u_{k}$, $v_{k}$ and$\left(u_{k},v_{k}\right)$, respectively.


(1) $$\rho_{uv}^{\left(k\right)}=\frac{m_{uv}^{\left(k\right)}}{m}-\frac{m_{u}^{\left(k\right)}}{m}\cdot \frac{m_{v}^{\left(k\right)}}{m},$$

where *m* is the number of cells in the dataset, $\frac{m_{u}^{\left(k\right)}}{m}$and$\frac{m_{v}^{\left(k\right)}}{m}$are the marginal probabilities of $lncR_{u}$and$mR_{v}$, respectively, and $\frac{m_{uv}^{\left(k\right)}}{m}$ is the joint probability of $lncR_{u}$ and $mR_{v}$. Empirically, we set $\frac{m_{u}^{\left(k\right)}}{m}=\frac{m_{v}^{\left(k\right)}}{m}=0.1$ as suggested in the CSN method. The statistic $\rho_{uv}^{\left(k\right)}$ can be normalized as follows:


(2) $${\hat{\rho }}_{uv}^{\left(k\right)}=\frac{\rho_{uv}^{\left(k\right)}-\mu_{uv}^{\left(k\right)}}{\sigma_{uv}^{\left(k\right)}}=\frac{\sqrt{m-1}\cdot \left(m\cdot m_{uv}^{\left(k\right)}-m_{u}^{\left(k\right)}m_{v}^{\left(k\right)}\right)}{\sqrt{m_{u}^{\left(k\right)}m_{v}^{\left(k\right)}\left(m-m_{u}^{\left(k\right)}\right)\left(m-m_{v}^{\left(k\right)}\right)}},$$

where$\mu_{uv}^{\left(k\right)}=0$ and $\sigma_{uv}^{\left(k\right)}=\sqrt{\frac{m_{u}^{\left(k\right)}m_{v}^{\left(k\right)}\left(m-m_{u}^{\left(k\right)}\right)\left(m-m_{v}^{\left(k\right)}\right)}{m^{4}\left(m-1\right)}}$ are the mean value and standard deviation for the statistic$\rho_{uv}^{\left(k\right)}$, respectively. ${\hat{\rho }}_{uv}^{\left(k\right)}$ follows standard normal distribution ${\hat{\rho }}_{uv}^{\left(k\right)}∼N\left(0,1\right)$. To evaluate the association significance of $lncR_{u}$ and $mR_{v}$, we obtain *p*-value corresponding to each $\rho_{uv}^{\left(k\right)}$. Smaller *p*-value indicates that $lncR_{u}$ is more likely to interact with $mR_{v}$ in cell *k*. Here, the cutoff of significance *p*-value is set to 0.05.

In cell *k*, there is an association between $lncR_{u}$and $mR_{v}$ if the significance *p*-value is less than 0.05. As a result, 276 cell-specific lncRNA-mRNA regulatory networks have been constructed. Here, each cell-specific lncRNA-mRNA regulatory network is a bipartite graph, where lncRNAs or mRNAs are nodes, and the pointing relationship from a lncRNA to a mRNA is represented by edges.

### 2.3. Inferring cell developmental stage-specific lncRNA regulation

Based on the identified 276 identified cell-specific lncRNA-mRNA regulatory networks, we can further infer cell developmental stage-specific lncRNA regulation. For example, we integrated all of cell-specific lncRNA-mRNA regulatory networks across 26 cells in GW16 to infer cell developmental stage-specific lncRNA regulatory network specific to GW16. Similarly, we can also infer cell developmental stage-specific lncRNA regulatory networks specific to GW19.5, GW20.5, GW21 and GW23.5. After integrating the identified cell-specific lncRNA-mRNA regulatory networks across all of single-cells in each cell development stage (GW16, GW19.5, GW20.5, GW21, and GW23.5), we can obtain five cell developmental stage-specific lncRNA-mRNA regulatory networks.

### 2.4. Network and functional analysis of cell developmental stage-specific lncRNA regulation

In this section, to further understand lncRNA regulation in the developing human neocortex, we conduct network and functional analysis of the identified cell developmental stage-specific lncRNA regulation.

#### 2.4.1. Network analysis of lncRNA regulation

The regulation for some lncRNAs is “on” in a biological condition whereas the regulation of some lncRNAs is “off” (Peng et al., 2017; Zhang J. et al., 2019). To unveil the commonality and heterogeneity between five cell development stages, we further infer the conserved and rewired lncRNA-mRNA regulatory networks and hub lncRNAs across five cell development stages. Previous studies (Hahn and Kern, 2005; Song and Singh, 2013) have shown that the essential nodes consists of nearly 20% of the nodes from a biological network. Therefore, the top 20% of lncRNAs with the largest node degrees in a cell developmental stage-specific lncRNA regulatory network are viewed as hub lncRNAs. In this work, the lncRNA-mRNA interactions or hub lncRNAs are “on” at five cell development stages, they are regarded as conserved interactions or hubs. If the lncRNA-mRNA interactions or hub lncRNAs are only “on” at a cell development stage, they are regarded as rewired interactions or hubs. Moreover, to show the commonality and heterogeneity across five cell development stages, we also calculate the similarity of five cell development stages in terms of lncRNA-mRNA interactions or hub lncRNAs by using the method in (Zhang et al., 2018).

#### 2.4.2. Functional enrichment analysis of lncRNA regulation

To evaluate the identified cell developmental stage-specific lncRNA regulation in five development stages (GW16, GW19.5, GW20.5, GW21, and GW23.5), we perform functional enrichment analysis of lncRNA regulation across different cell development stages. Moreover, we utilize the experimentally validated lncRNA-target interactions from NPInter v4.0 (Teng et al., 2020), LncTarD (Zhao et al., 2020) and LncRNA2Target (Cheng et al., 2019) for validation. Since the human neocortex cells are highly correlated with autism spectrum disorder (ASD), we use a list of lncRNAs and mRNAs associated with ASD to further explore ASD-related lncRNA regulation. The list of ASD-related lncRNAs and mRNAs are from previous studies (Ziats and Rennert, 2013; Wang et al., 2015; Cogill et al., 2018) and Simons Foundation Autism Research Initiative (SFARI) v2.0 (Abrahams et al., 2013).

To uncover potential biological functions related to the identified cell developmental stage-specific lncRNA regulation, we use the clusterProfiler R package (Yu et al., 2012) to perform Gene Ontology (GO) (Ashburner et al., 2000), Kyoto Encyclopedia of Genes and Genomes (KEGG) (Kanehisa and Goto, 2000), Reactome (Fabregat et al., 2018), Hallmark (Subramanian et al., 2005), CellMarker (Zhang X. et al., 2019) enrichment analysis. If the adjusted *p*-value (adjusted by Benjamini–Hochberg method) of these GO, KEGG, Reactome, Hallmark and CellMarker terms is less than 0.05, we regard them as significantly enriched terms. In addition, to evaluate whether the lncRNAs and mRNAs in each human neocortex development stage are significantly enriched in ASD or not, we use a hyper-geometric test to conduct ASD enrichment analysis. For each cell developmental stage-specific lncRNA regulatory network, the significance *p*-value enriched in ASD is calculated as follows:


(3) $$p-value=1-\overset{r-1}{\underset{t=0}{\sum }}\frac{\left(\begin{array}{c} S\\ t \end{array}\right)\left(\begin{array}{c} N-S\\ M-t \end{array}\right)}{\left(\begin{array}{c} N\\ M \end{array}\right)},$$

where *N* represents the number of genes in the dataset, *S* is the number of ASD-related genes in the dataset, *M* and *r* are the number of genes and ASD-related genes in each cell developmental stage-specific lncRNA regulatory network, respectively. We set the cutoff of *p*-value to 0.05.

## 3. Results

### 3.1. The lncRNA regulation is unique across development stages of human neocortex

To investigate the lncRNA regulation across development stages of human neocortex, we have constructed five cell developmental stage-specific lncRNA-mRNA regulatory networks. Based on the identified cell developmental stage-specific lncRNA-mRNA regulatory networks in each development stage (GW16, GW19.5, GW20.5, GW21, and GW23.5), we further explore the uniqueness of the lncRNA regulation in terms of cell developmental stage-specific lncRNA-mRNA interactions and hub lncRNAs.

**Figure 2:**
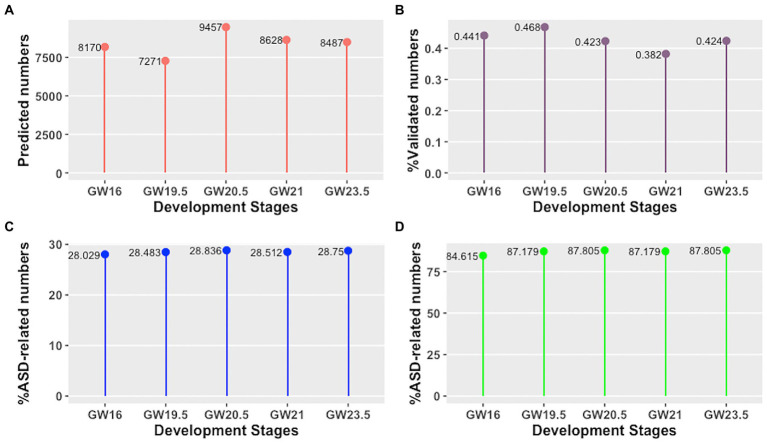
Cell developmental stage-specific lncRNA-mRNA interactions and hub lncRNAs in different human neocortex development stages. **(A)** Number of predicted lncRNA-mRNA interactions. **(B)** Percentage of validated lncRNA-mRNA interactions. **(C)** Percentage of ASD-related lncRNA-mRNA interactions. **(D)** Percentage of ASD-related hub lncRNAs.

Firstly, we have explored the identified cell developmental stage-specific lncRNA-mRNA interactions and hub lncRNAs using the following four aspects: (i) the number of predicted lncRNA-mRNA interactions, (ii) the percentage of validated lncRNA-mRNA interactions, (iii) the percentage of ASD-related lncRNA-mRNA interactions, and (iv) the percentage of ASD-related hub lncRNAs. As shown in Figures 2A,B, we find that the number of predicted lncRNA-mRNA is different across different development stages of human neocortex, as well as the percentage of validated lncRNA-mRNA. Moreover, the percentage of ASD-related lncRNA-mRNA interactions and hub lncRNAs is also different across different development stages of human neocortex (see Figures 2C,D). This result shows that the lncRNA regulation in each human neocortex development stage is different. Furthermore, we investigate the conserved and rewired lncRNA-mRNA interaction and hub lncRNAs across different human neocortex development stages. Here, the conserved lncRNA-mRNA interactions or hub lncRNA are “on” at five cell development stages and the rewired lncRNA-mRNA interactions or hub lncRNAs are only “on” at a cell development stage. We discover that the percentage of the conserved and rewired interactions is 53.34% (5,907 out of 11,074) and 13.57% (1,503 out of 11,074), respectively. The remaining interactions (33.09%) are only “on” at two to four cell development stages. Accordingly, this suggests that more than half of the lncRNA regulation may tend to be conserved across five human neocortex development stages. The detailed information of the conserved and rewired lncRNA-mRNA interactions and hub lncRNAs can be seen in Supplementary material 1.

**Figure 3:**
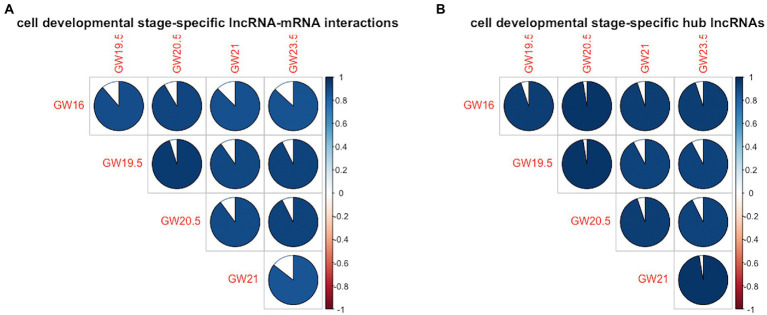
Cell development stage similarity plot across five human neocortex development stages. **(A)** Similarity plot in terms of cell developmental stage-specific lncRNA-mRNA interactions. **(B)** Similarity plot in terms of cell developmental stage-specific hub lncRNAs. Colored areas indicate higher similarity between human neocortex development stages.

In terms of the similarity of the lncRNA-mRNA interactions between the identified cell developmental stage-specific lncRNA-mRNA regulatory networks, the range of cell-development similarity is [0.85, 0.95]. As shown in Figure 3A, the cell development stage similarity is less than 1 between any pair of the human neocortex development stages. In terms of the similarity of the hub lncRNAs between the identified cell developmental stage-specific lncRNA-mRNA regulatory networks, the range of cell development stage similarity is [0.92, 0.97]. As shown in Figure 3B, the cell development stage similarity is also less than 1 between any pair of the human neocortex development stages. To show the difference of lncRNA regulation, we calculate the similarity of lncRNA-mRNA interactions and hub lncRNAs between any pairs of developmental stages. Here, we focus on the similarity differences rather than similarity. Accordingly, in Figure 3, these similarity differences (dissimilarity) show that the lncRNA regulation is always different between any pair of development stages.

In summary, in terms of cell developmental stage-specific lncRNA-mRNA interactions and hub lncRNAs, the lncRNA regulation between any pair of cell development stages is always different, indicating the uniqueness of lncRNA regulation in each human neocortex development stage.

### 3.2. The cell developmental stage-specific regulation of lncRNA biomarkers across human neocortex development stages

To further investigate the lncRNA regulation across different development stages of human neocortex, we conduct a case study to explore the cell developmental stage-specific regulation of 18 lncRNA biomarkers (Liu S. et al., 2016). The 18 lncRNA biomarkers are grouped into 7 known cell types: endothelia (*LINC00339*, *TRIM52-AS1*), radial glia (*LINC00943*, *MAGI2-AS3*, *RUSC1-AS1*), dividing radial glia (*THAP9-AS1*), intermediate progenitors (*DGCR11*), newborn neurons (*INHBA-AS1*, *MYT1L-AS1*, *KIF9-AS1*), maturing excitatory (*MIR137HG*, *PWAR6*, *SIK3-IT1*, *NAV2-AS3*, *DAPK1-IT1*), inhibitory interneurons (*DLX6-AS1*, *SOX2-OT*, *MEG3*). Previous studies (Wang et al., 2015; Liu S. et al., 2016; Cogill et al., 2018; Li and Guo, 2020) have shown that they play important roles in cell cycle, cell proliferation, ASD and several other key biological functions.

**Figure 4:**
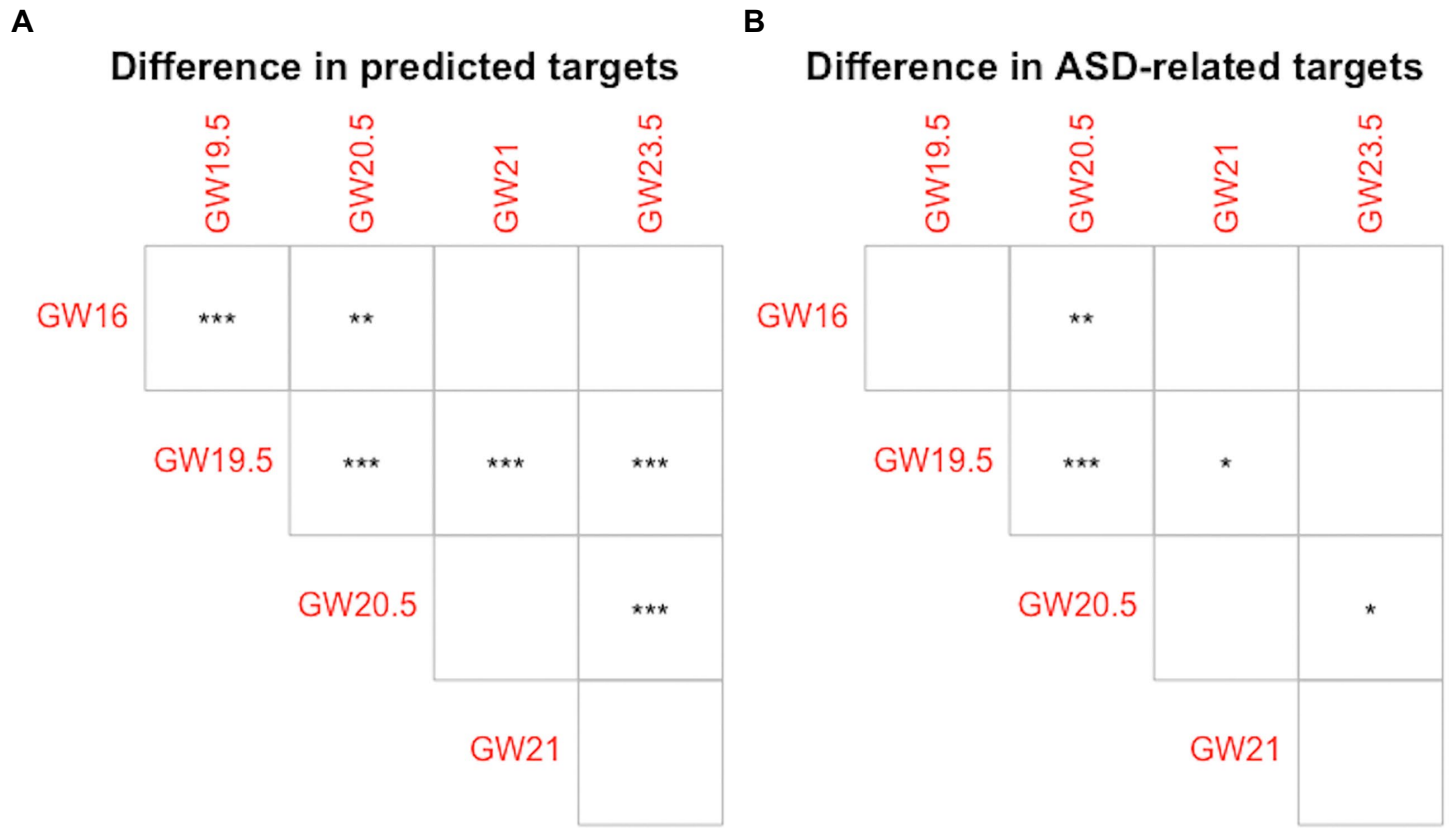
The cell developmental stage-specific regulation of lncRNA biomarkers. **(A)** Difference in predicted targets of lncRNA biomarkers. **(B)** Difference in ASD-related targets of lncRNA biomarkers. Empty square shape denotes *p*-value > 0.05, * denotes *p*-value < 0.05, **denotes *p*-value < 0.01, and *** denotes *p*-value < 0.001.

To measure the difference in the cell developmental stage-specific regulation of lncRNA biomarkers between each pair of human neocortex development stages, we compare the distributions of the number of predicted targets, and the distributions of the percentage of ASD-related targets of lncRNA biomarkers in different human neocortex development stages by using a two-sample Kolmogorov–Smirnov (KS) test (Conover, 1999). The non-parametric KS test can be used to assess whether the distribution of the number of predicted targets, or the percentage of ASD-related targets of lncRNA biomarkers in one human neocortex development stage is significantly shifted compared with the distribution in another human neocortex development stage. To obtain these distributions, we calculate the number of predicted targets and the percentage of ASD-related targets of lncRNA biomarkers, respectively, in each human neocortex development stage. In Figure 4, we show the difference of any pairs of cell development stage in terms of predicted targets and ASD-related targets of 18 known lncRNA biomarkers. As shown in Figure 4A, in the case of predicted targets, the regulations of lncRNA biomarkers between six pairs of five human neocortex development stages are significantly different (*p*-value < 0.01). In Figure 4B, in the case of ASD-related targets, the regulations of lncRNA biomarkers between four pairs of five human neocortex development stages are significantly different (*p*-value < 0.05). These results display that the regulation of lncRNA biomarkers may be cell developmental stage-specific. To illustrate the role of broad lncRNA regulation across different development stages, we make the following comparison analysis. In the broad transcriptomic dysregulation study related to ASD (Gandal et al., 2022), the experiment results show that cell-type-specific gene expression changes correspond to most of the changes in bulk tissue gene expression. This also indicates that cell-type-specific changes (small range level) explain widespread molecular changes across the cerebral cortex in ASD. In our study, all inferred lncRNA-mRNA interactions are cell developmental stage-specific. For ASD-related targets, the regulation of lncRNA biomarkers is also cell developmental stage-specific. This may indicate that lncRNA biomarkers regulation (small range level) also explain widespread lncRNA regulation across different human neocortex development stages. In addition, we also observe that the number of conserved targets of lncRNA biomarkers is larger than the number of rewired targets of them (see Figure S1 in Supplementary material 2). These differences indicate that the dominant lncRNA regulation type across human neocortex development stages may be conserved lncRNA regulation for lncRNA biomarkers. The detailed information of the conserved and rewired targets of lncRNA biomarkers can be seen in Supplementary material 3.

For the validated lncRNA-mRNA interactions across development stages, we explore the connections between memory epigenetic regulation and validated lncRNAs by regulating gene expression. In the previous study (Irwin et al., 2021), we obtained a set of lncRNAs that are related to the memory epigenetic regulation. By comparing these memory-related lncRNAs with the validated lncRNA-mRNA interactions, we find four lncRNAs (*GAS5*, *NEAT1*, *MALAT1*, *MEG3*), where *MEG3* is a biomarker of inhibitory interneurons. *GAS5* and *MALAT1* have a close association with Parkinson’s disease and aging and are involved in neurodegeneration (Irwin et al., 2021). *GAS5* regulates novelty induced behavior and *MALAT1* regulates gene expression and synapse formation to improve synapse development. *MEG3* and *NEAT1* are related with Alzheimer’s disease and are involved in cognitive decline (Irwin et al., 2021). *MEG3* modulates mHTT aggregation and *NEAT1* provides neuroprotection against oxidative stress induced injury. This shows that lncRNA regulation unveiled by CDSlncR plays an important role on epigenetic regulation.

In general, lncRNAs perform a specific biological function through regulating target genes. Since the conserved lncRNA regulation is likely to be the dominant regulation of lncRNA biomarkers across human neocortex development stages, we further perform functional enrichment analysis of the conserved lncRNA-mRNA interactions of lncRNA biomarkers. Functional enrichment analysis show that the conserved lncRNA-mRNA interactions of lncRNA biomarkers are significantly enriched in several terms of Gene Ontology (GO), Kyoto Encyclopedia of Genes and Genomes Pathway (KEGG), Reactome, Hallmark, CellMarker and ASD (see Table S1 in Supplementary material 2). In addition, several significant terms including the GO biological process “mRNA catabolic process” (Wang et al., 2020), “regulation of mRNA metabolic process” (Chiocchetti et al., 2014), KEGG pathway “mRNA surveillance pathway” (Addington et al., 2011), Reactome pathway “mRNA Splicing” (Parras et al., 2018), Hallmark “HALLMARK_MYC_TARGETS_V1” (Jin et al., 2018), and “Embryonic prefrontal cortex, Normal, Interneuron” (Marín, 2012) are closely associated with ASD. These results indicate that the conserved lncRNA-mRNA interactions of lncRNA biomarkers are functional across different human neocortex development stages. The detailed enrichment analysis results of the conserved lncRNA-mRNA interactions can be seen in Supplementary material 4.

### 3.3. CDSlncR is effective in predicting cell developmental stage-specific lncRNA targets across different human neocortex development stages

**Figure 5:**
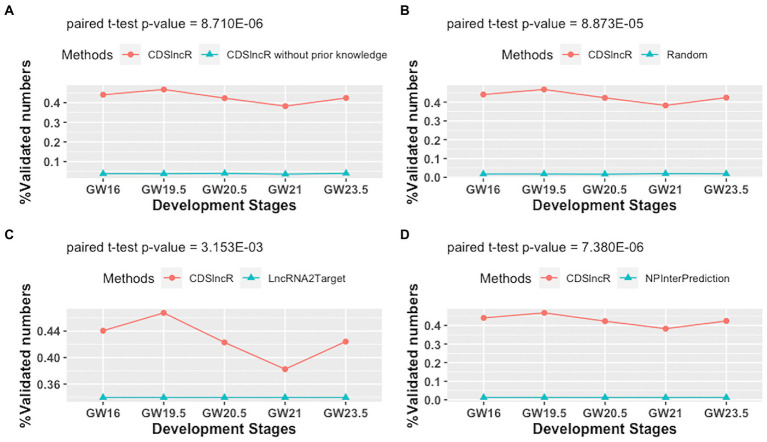
Comparison in terms of the percentage of validated lncRNA-mRNA interactions. **(A)** Comparison results between CDSlncR (with prior knowledge) and CDSlncR (without prior knowledge). **(B)** Comparison results between CDSlncR and Random method. **(C)** Comparison results between CDSlncR and LncRNA2Target. **(D)** Comparison results between CDSlncR and NPInterPrediction.

To assess the effectiveness of CDSlncR, we compare it with the other four methods (CDSlncR without using prior knowledge, Random method, LncRNA2Target (Cheng et al., 2019), and NPInterPrediction (Teng et al., 2020)) in terms of the percentage of validated lncRNA-mRNA interactions across five human neocortex development stages. Here, NPInterPrediction denotes the prediction type of lncRNA-target interactions from NPInter (Teng et al., 2020). Random method generates lncRNA-mRNA interactions by randomly selecting lncRNAs and mRNAs from human neocortex dataset. In Figure 5A, the comparison result demonstrates the effectiveness of prior knowledge for the accuracy of lncRNA target prediction. Moreover, in Figure 5B, the paired t-test shows that the percentage of validated lncRNA-mRNA interactions across five human neocortex development stages by CDSlncR is larger than that of the random method at a significant level (*p*-value = 8.873E−05). Next, we compare the proposed CDSlncR with LncRNA2Target. As displayed in Figure 5C, the result using a paired t-test indicates that the percentage of validated lncRNA-mRNA interactions across five human neocortex development stages by CDSlncR is also larger than that of LncRNA2Target at a significant level (*p*-value = 3.153E−03). Finally, we compare the proposed CDSlncR with NPInterPrediction. As shown in Figure 5D, the result using a paired t-test indicate that the percentage of validated lncRNA-mRNA interactions across five human neocortex development stages by CDSlncR is also larger than that of NPInterPrediction at a significant level (*p*-value = 7.380E−06). Altogether, these comparison results show that CDSlncR is effective in predicting cell developmental stage-specific lncRNA targets across different human neocortex development stages.

## 4. Conclusion

As is known, lncRNA regulation is important in many biological processes including RNA silencing, transcriptional regulation of gene expression, ASD transcriptional regulation, cellular functions, signaling pathways, and neurodegenerative and neuropsychiatric diseases. Previous studies (Shi et al., 2016; Statello et al., 2021) have shown that lncRNA regulation is condition-specific. In this work, to understand lncRNA regulation specific to each human neocortex development stage, we propose a novel method CDSlncR to infer cell developmental stage-specific lncRNA regulation. Since we study cell developmental stage-specific lncRNA regulation by incorporating prior knowledge, the proposed CDSlncR is a supervised method.

As for CDSlncR, there are some limitations to be improved in future. Firstly, the accuracy of predicted cell developmental stage-specific lncRNA-targets can be further increased by incorporating more high-confidence prior knowledge of lncRNA-target interactions. Secondly, the identified cell developmental stage-specific lncRNA-mRNA regulatory networks are correlation networks rather than causal networks. It is our future plan to infer cell developmental stage-specific lncRNA causal regulatory networks. Thirdly, CDSlncR only considers lncRNAs as regulators where lncRNAs directly regulate the expression of mRNAs, and has not considered lncRNAs as competing endogenous RNAs (ceRNAs). The ceRNA hypothesis (Salmena et al., 2011) indicates that lncRNAs can act as ceRNAs to influence the expression of mRNAs. Therefore, we also plan to infer cell developmental stage-specific lncRNA-related ceRNA networks in future. Finally, CDSlncR is only applied into a small-scale single-cell transcriptomics data for studying cell developmental stage-specific lncRNA regulation. In the future, we will apply CDSlncR into large-scale single-cell RNA sequencing datasets in the other human cortical regions, including frontal cortex, prefrontal cortex, temporal lobe, etc.

Although there are some limitations as discussed above, CDSlncR is still useful for investigating the heterogeneity of lncRNA regulation across different human neocortex development stages. Especially, CDSlncR can be applied into the study of brain disorders (Liu S. et al., 2016; Cogill et al., 2018), where a few cells could be profiled. Altogether, we believe that CDSlncR can be an effective method to enhance the non-coding RNA (e.g., lncRNA) research for biologists.

## Data availability statement

The datasets presented in this study can be found in online repositories. The names of the repository/repositories and accession number(s) can be found in the article/Supplementary material.

## Author contributions

JZ conceived and supervised the project. JZ and MH contributed to conception and design of the study. MH and JM wrote the manuscript. MH and JZ reviewed and edited the manuscript. All authors contributed to the article and approved the submitted version.

## Funding

This work was supported by JST SPRING (JPMJSP2124), the National Natural Science Foundation of China (61963001), and the Yunnan Fundamental Research Projects (202001AT070024 and 202101BA070001-221).

## Conflict of interest

The authors declare that the research was conducted in the absence of any commercial or financial relationships that could be construed as a potential conflict of interest.

## Publisher’s note

All claims expressed in this article are solely those of the authors and do not necessarily represent those of their affiliated organizations, or those of the publisher, the editors and the reviewers. Any product that may be evaluated in this article, or claim that may be made by its manufacturer, is not guaranteed or endorsed by the publisher.

## Footnotes

^1^ https://www.genenames.org/
