# Supplementary material for: Inferring cell developmental stage-specific lncRNA regulation in the developing human neocortex with CDSlncR
Source: Front Mol Neurosci. 2023 Jan 13;15:1037565. doi: 10.3389/fnmol.2022.1037565 (PMC9880432; doi:10.3389/fnmol.2022.1037565)
Supplement: Supplementary file 2 [file Data_Sheet_1.docx]

Supplementary Material

# Supplementary Figures


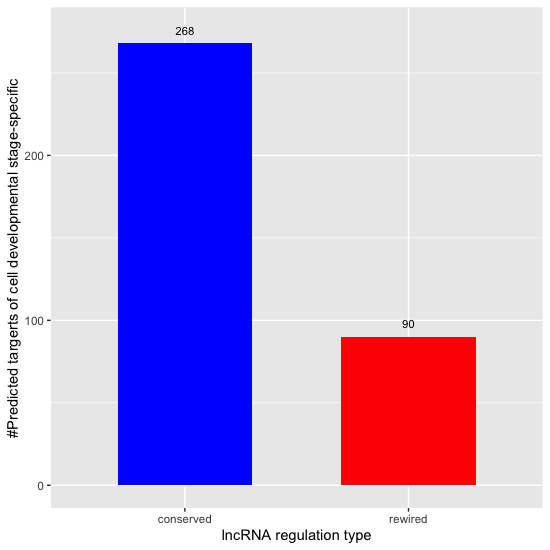


**Supplementary Figure S1.** Number of conserved and rewired targets of cell developmental stage-specific lncRNA regulation among different human neocortex development stages.

# Supplementary Tables

**Supplementary Table S1.** Enrichment analysis of the conserved lncRNA-mRNA interactions of lncRNA biomarkers.

| Number of stages | #GO | #KEGG | #Reactome | #Hallmark | #CellMarker | Enriched in ASD or not? |
| --- | --- | --- | --- | --- | --- | --- |
| 5 | 130 | 2 | 40 | 6 | 2 | Yes |
